# Implicit Sexual Cognitions in Women with Ambiphilic Sexual Attractions: A Comparison to Androphilic and Gynephilic Women

**DOI:** 10.1007/s10508-023-02727-y

**Published:** 2023-11-06

**Authors:** Robert J. Snowden, Nicola S. Gray, Katie S. Uzzell

**Affiliations:** 1 School of Psychology, Cardiff University, Cardiff, CF10 3AT Wales UK; 2 Department of Psychology, Swansea University, Swansea, Wales UK; 3 Swansea Bay University Health Board, Bridgend, UK

**Keywords:** Bisexual, Ambiphilia, Gynephilia, Androphilia, Implicit Association Test, Sexual orientation

## Abstract

Previous research using indirect cognitive measures (sometimes referred to as implicit measures) of sexual attraction have shown that women who are attracted to men (androphilic women) show category non-specific responses, whereas those who are attracted to women (gynephilic) show a category-specific bias to women. The purpose of the present study was to examine whether women who explicitly report approximately equal attraction to men and women (ambiphilic) would show similar non-category specific attraction at this implicit level or whether their responses would be more similar to those of gynephilic women. An implicit association task and a priming task were given to 169 women alongside measures of their self-labelled sexual orientation and an explicit measure of their sexual attraction to men and women. The results replicated previous findings of little bias towards either gender in androphilic women and of a strong bias towards females in gynephilic women. The ambiphilic women also showed a strong bias towards females. The findings clearly show that early automatic associations to sex are biased towards females in ambiphilic women and are not consistent with their explicit statements of preference.

## Introduction

Measurement of sexual attraction is fundamental part of research into human sexuality, though people’s sexual identity, behavior, and sexual attractions do not necessarily go hand in hand (Colledani & Ciani, 2021; Zivony, 2020). Recently, there has been the development of a variety of techniques that aim to supplement people’s explicit statement of sexual attraction by use of indirect measures that look at a person’s immediate associations or attention to sexual stimuli. Such measures are often termed “implicit” measures in that they aim to measure automatic evaluations of a stimulus to which the person may or may not be aware (see Vianello & Bar-Anan, 2021 for a discussion of this point).

The information processing model (IPM; Janssen et al., 2000) proposes that there are two cognitive pathways for the processing of sexual stimuli. The first, which they term “appraisal,” is a pre-attentive and largely unconscious detection of the sexual nature of the stimulus which matches the stimulus features with memory elements and produces both genital responses and automatic attentional engagement. The second pathway, which they term “response generation” is more a later controlled processing that elaborates the sexual meaning of the stimulus and gives rise to affective/subjective feelings of sexual arousal as well as contributing further to genital arousal. This may also direct attention to the stimulus in a controlled manner. As such, indirect/implicit measures may be able to measure the “appraisal” processes such as the automatic associations produced by the stimulus, and any initial shifts in attention. On the other hand, people’s explicitly stated sexual interests are likely to reflect the second pathway of “response generation.” Hence, it is possible that measures that are reliant on the first appraisal pathway may produce results that are inconsistent with measures that are reliant on the second response generation pathway. We suggest that some indirect measures (see review below) reflect these early appraisal processes, whereas direct (or explicit) measures reflect the controlled response generation processes.

Chivers (2017) (see also Chivers et al., 2004, 2007, 2010) has shown that genital responses to sexual stimuli are not-category specific for androphilic (heterosexual) women, but are category specific for gynephilic (homosexual) women (with both androphilic (homosexual) and gynephilic (heterosexual) men showing category specific arousal as well). Further, similar studies using ambiphilic (bisexual) women have shown that their genital responses are also category specific with greater arousal being caused by images of females in comparison to male (Bouchard et al., 2015; Chivers et al., 2015; Timmers et al., 2015). However, interpretation of these results in terms of the IPM (Janssen et al., 2000) or other dual-process models is difficult. The stimuli in such studies are video or auditory stimuli that last for many seconds (as do the measurement variables) and so would activate both the early automatic processes and the later controlled processes. Hence, it is not possible to disentangle the contribution of each.

Studies using indirect cognitive measures of sexual attraction have also shown that in most cases these indirect techniques correspond well with the person’s explicitly stated attractions (e.g., Snowden et al., 2008). However, some studies have shown this not to be the case for androphilic women. For instance, Snowden and Gray (2013) showed that androphilic women had similar levels of sexual associations to men and to women. In contrast, gynephilic women showed strong sexual associations to women. However, at present, there are few data related to ambiphilic women using indirect cognitive measures. Given that we expect their explicit statements of sexual attraction to be approximately the same to both men and women, we envisaged three possible patterns of results. First, their implicit sexual attractions could be approximately equal to both male and female stimuli, which is in-line with their explicit judgement, and is similar to the implicit attractions of androphilic women. Second, their implicit attractions may be much stronger to female stimuli than to male stimuli. This would be different to their explicitly stated sexual interest, but would be similar to the implicit attractions of gynephilic women. Finally, their implicit attractions may be stronger to females than those of androphilic women, but less than those of gynephilic women. In this case, the indirect measure of sexual attraction would be inconsistent with their explicit judgements but would lie between the implicit sexual associations of androphilic and gynephilic women.

### Previous Research Related to Ambiphilic Women

A variety of techniques have been developed that aim to examine sexual attraction. In this section, we will limit coverage to those that have used purely cognitive techniques and thus do not cover physiological techniques such as genital responses (see Bouchard et al., 2015; Chivers et al., 2004), pupillometry (see Rieger et al., 2015; Snowden et al., 2019) or eye-tracking methods (see Dawson et al., 2017; Rupp & Wallen, 2007).

#### Viewing Time

Perhaps the most used implicit method in assessing sexual attraction is that of viewing time (Israel & Strassberg, 2009). In the standard Viewing Time paradigm, the participant is given a series of pictures of people to observe and rate on some dimension (such as the attractiveness of the person in the image which can then be used as an explicit measure of attraction). The amount of time taken viewing the image is then taken as a proxy for how attractive they found the person. Lippa (2013) examined both ambiphilic women and men using this viewing time paradigm. Lippa found that both these groups viewed stimuli of men and women for approximately the same amount of time, thus supporting the idea that their implicit attractions are commensurate with their explicit statements. For androphilic women viewing times were greater for male stimuli, while gynephilic women viewed the female stimuli for longer. Hence, the response of the ambiphilic women were between those of the androphilic and gynephilic women. Similar results were found in the studies of Ebsworth and Lalumière (2012) and Rullo et al. (2010). In a further large-scale on-line study, Lippa (2017) replicated most of these results, however, in this sample the ambiphilic women did show slightly greater viewing times to the female stimuli. Most viewing time studies involve the person looking at the stimuli for a “long” time (on the order of seconds). As such, this paradigm allows for the controlled processes (response generation) of the IPM (Janssen et al., 2000) to influence responding and does not isolate the early automatic evaluation of the stimulus (appraisal).

#### Implicit Association Test

The Implicit Association Test (IAT) involves the simultaneous categorization of two sets of stimuli and has been widely used in many areas of psychology (Greenwald et al., 1998). It relies on the idea that if two stimuli are associated within the mind of a person it will be easy to categorize them onto the same response, whereas it will be hard to categories them into different responses. A such, the paradigm is thought to capture early automatic associations that are produced by the categorization of the stimulus, and could provide a measure of the sexual appraisal process (Janssen et al., 2000). The task has been developed to look at pedophilic sexual associations (Gray et al., 2005) and is able to distinguish child-sex offenders from non-offenders with “large” effect sizes (Babchishin et al., 2013). Snowden et al. (2008) further developed the task to examine sexual associations in gynephilic and androphilic men. They found that task could distinguish between these groups with high accuracy (AUC = 0.92). Hence, the task has a strong pedigree for use in measuring sexual interest.

In a series of IAT studies, it has been shown that men’s associations are in close accord with their explicit statements of attraction, with gynephilic men showing strong associations between female images and sex, and androphilic men showing strong associations between male images and sex (Snowden & Gray, 2013; Snowden et al., 2008, 2020). Ambiphilic men showed no significant difference in their sexual associations to male and female categories (Snowden et al., 2020). Using modified versions of the IAT that attempted to measure implicit sexual attraction to male image and female images separately, Snowden et al. (2020) showed that ambiphilic men had sexual attraction to both male and female categories (compared to “neutral”) with approximately equal magnitude. Therefore, their implicit sexual attractions are consistent with the explicit sexual attraction and with their self-reported sexual orientation. For women, gynephilic women show a strong attraction to female image, but androphilic women show approximately similar attraction to both the male and female images (Snowden & Gray, 2013). Hence, for andophilic and gynephilic women, their implicit sexual attractions are in accord with findings from studies of genital arousal (e.g., Chivers et al., 2004). There has been no study of the implicit sexual attraction for ambiphilic women.

Kirby et al. (2021) did use an IAT in a sample of ambiphilic women. However, their tasks aimed to examine implicit self-identity (whether a person sees themselves as ambiphilic, androphilic or gynephilic) by having people classify pictures, for example, as depicting either “same-sex” or “different-sex” relationships, and classifying words as either “self” or “others.” They demonstrated that these implicit identities corresponded well with their self-reported sexual identities.

#### Priming Task

In the priming task, the participant must make a speeded categorization of a target stimulus. However, just before this target is presented, a prime stimulus is presented that is not part of the response set. This prime stimulus, however, can alter the speed of the response to the target stimulus (Fazio & Olson, 2003). Again, this paradigm appears to be reliant on early automatic associations or response biases created by the stimulus and could be used as a measure of the appraisal process (Janssen et al., 2000).

Snowden et al. (2008) developed a version of the task that had participants categorize words as either sex or not-sex, and used primes of either pictures of men or women that were presented very briefly (200 ms) just before the target word. In the context of a measure of sexual interest we might expect to see that stimuli that the person finds sexually attractive will prime the response of “sex.” Hence, responses to the sex words will be faster as they are congruent with this appraisal of the prime stimulus, while responses to the non-sex words would be slower as they are incongruent with the appraisal of the prime stimulus. For gynephilic men, the images of women speeded the response to sex words, but for androphilic men the images of men speeded the response to the sex words.

The task distinguished between these two groups with a high degree of accuracy (AUC = 0.86) suggesting it is a valid instrument for measuring sexual attraction. The task has also been applied to ambiphilic men (Snowden et al., 2020) who showed an approximately equal priming from both male and female images whereas the gynephilic men were primed by the female stimuli, and the androphilic men by the male stimuli, replicating the results of the previous study (Snowden et al., 2008).

Snowden and Gray (2013) used the priming technique to examine sexual interest in both androphilic and gynephilic women. In line with the notion that androphilic women do not show category-specific sexual interest (Chivers, 2017; Chivers et al., 2004), there was no significant difference in the priming effect produces by male and female sexual images. However, for the gynephilic women, the female images produced strong priming effects whereas the male images did not. However, this task has not been used to assess sexual attractions in ambiphilic women.

While the IAT and the priming task share many similarities, they appear to assess different stages of processing. Indeed, correlations between measures of implicit biases using the two tasks are weak (Olson & Fazio, 2003) suggesting they tap different psychological constructs. The priming effect is driven by the nature of the exemplar being used as a prime. For instance, Livingston and Brewer (2002) showed that “prototypical” black faces produced a greater priming effect than black faces that were judged as less prototypical. On the other hand, the IAT appears to arise at a later stage after the categorization of the target stimulus into one of the target categories. Hence, the actual exemplars used should be largely irrelevant to the IAT effect. As an example, De Houwer (2001) showed that the perceived valence of the target (good or bad) had little effect on an IAT designed to measure prejudice towards/against British vs Germans—see also Mitchell et al. (2003). In terms of sexual processing, this means that the priming task should access the sexual associations triggered by the actual exemplar presented (whether the person finds that particular image to be of sexual interest), whereas the IAT accesses whether the category that particular image represents triggers sexual associations.

The present study examined the automatic sexual attraction using both the IAT and the prime tasks in ambiphilic women to see if these cognitions were in accord with their explicit statements. Data were also collected from androphilic and gynophilic women for replication and comparison purposes. We predicted that the implicit sexual attraction of ambiphilic women would be towards the female images (and therefore different from their explicit reports) but would lie between the strong female attraction of gynephilic women and the non-categorical attraction of androphilic women.

## Method

### Participants

The study was conducted at two sites in order to obtain a large sample of women with a range of self-reported sexual orientations.

The Cardiff sample was recruited from a range of advertisements using Facebook and Twitter. We also handed out leaflets and recruited participants from various events, including BiFest Wales, PrideCymru mardi gras, and the LGBT + Society of Cardiff University. We encouraged participants to inform their friends about the study. We did not advertise for one or more particular group of people, sexual interest, or orientation but stressed that we were interested in human sexuality and that we wished to test people of all sexual orientations. Both cisgender and transgender individuals were welcome to participate (though no transgender individuals participated in this study). The leaflets/advertisements asked for participants willing to take part in our studies. They stated that the studies would involve looking at images of a sexual nature and we would be asking them about their sexual interests and behaviors. People who agreed to be contacted gave contact details. They were then contacted to arrange a time to be tested. In all, 73 women were successfully recruited through this method. Their mean age was 24.2 years (SD 6.2, range 18–51) and with a mean Kinsey score (see below) of 2.3 years (SD 2.3, range 0–6). No other demographic information was taken.

The Swansea sample was recruited from a range of advertisements across the University campus and by using adverts on Facebook and Twitter asking for women to volunteer to participate in research investigating human sexuality. The adverts stated that the studies would involve looking at images of a sexual nature and we would be asking them about their sexual interests and behaviors. People who agreed to be contacted gave contact details. They were then contacted to arrange a time to be tested. In all, 96 women were successfully recruited through this method. Their mean age was 27.9 years (SD 9.8, range 18–56) and with a mean Kinsey score (see below) of 2.3 (SD 2.3, range 0–6). No other demographic information was taken.

For statistical analysis, we formed three groups. The androphilic group (*N* = 78, sample 1 = 29, sample 2 = 49) consisted of women who self-reported as being heterosexual and gave Kinsey ratings of 0 or 1. For the ambiphilic group (*N* = 48, sample 1 = 26, sample 2 = 22), we included people who self-reported as being bisexual and had Kinsey ratings of 2–4. The gynephilic group (*N* = 43, sample 1 = 25, sample 2 = 18) consisted of participants who self-reported as being homosexual and gave Kinsey ratings of 5 or 6.

### Procedure

The protocols and procedures for this study were similar to those described in Snowden et al. (2020). Before testing took place, participants were given a detailed information sheet that explained the nature of the research and questionnaires and that the data from the tasks would be kept confidentially. Participants were encouraged to ask questions about the tasks and procedures. They were shown a sheet of paper on which all the stimuli to be used were presented in “thumbnails” to make sure that they understood the exact nature of the stimuli they would be viewing. Participants then signed a consent form. We then asked them to fill out the demographic questionnaire that included questions about how they described themselves in terms of their sexuality, the Kinsey scale (Kinsey et al., 1948), and a feeling thermometer about their sexual interests. Participants then completed a battery of tests that looked at different aspects of their sexuality and included both physiological recordings and behavioral tasks. For the participants in the Cardiff sample, the sex-IATs were always presented as the second set of tasks in this battery (following pupillometry measurements). Among the sex-IATs, the male–female sex IAT was always presented first. The order of the other two sex-IATs (male-neutral sex IAT and female-neutral sex IAT) was randomized. The priming task was then presented. For the Swansea sample, the order was the same save the pupillometry task was not performed.

### Stimuli and Materials

#### Kinsey Scale

Sexual attraction was evaluated by a Kinsey scale (Kinsey et al., 1948) with seven options. Option 0 was labelled as “Exclusively attracted to the other gender,” option 3 was labelled as “Equally attracted to both genders,” and option 6 was labelled as “Exclusively attracted to the same gender.” The seventh option was an “*X*” and was labelled “non-sexual or other.”

#### Feeling Thermometer

Direct ratings of feelings toward the construct pairs “sex with men” and “sex with women” were obtained using the feeling thermometer, which employs the heuristic of a thermometer. Participants rated feelings from “cold/unfavorable” at 0 to “warm/favorable” at 100 by circling the appropriate number on the scale.

#### Implicit Association Tests

For the Cardiff sample, the tasks were undertaken in a laboratory on a computer screen 48 cm wide and 30 cm in height running at 60 Hz under the control of DirectRT software. The images were presented in the center of the screen and were 25 cm wide and 15 cm high. The words were presented in the center of the screen and had a letter size of 1 cm. Labels (e.g., “men or sex”) were placed in the upper right and left of the screen to serve as a reminder of the correct responses. Participants sat approximately 57 cm from the screen and used the keyboard to give their responses. For the Swansea sample, the tasks were presented on a laptop.

We represented the concept of “men” by the use of eight pictures of men (all pictures were taken from the International Affective Picture System (IAPS: Lang et al., 1997; IAPs nos.: 4460, 4470, 4490, 4503, 4520, 4534, 4550, 4561) and the concept “women” with eight pictures of women (IAPs nos.: 4002, 4003, 4141, 4142, 4210, 4232, 4235, 4240). The pictures all depicted a single person either nude or partially dressed. We made an approximate attempt to match the pictures according to pose, ethnicity, etc. but no formal measurements were made.

The concepts of “sex” and “not sex” were represented by words developed in pilot work (which included both offender and non-offender samples (see Gray et al., 2005). The sex words were: sex, fuck, lick, cum, cock, kiss, lust, and suck. The not-sex words were laugh, eye, toe, elbow, run, smile, walk, and knee.

The IAT contained two stages. In the first stage, participants classified pictures of men or sex words on the right button, and pictures of women and not-sex words on the left button. Participants were instructed to “Try and respond as fast as you can, without making many errors.” Fifty-six trials (28 pictures and 28 words) were then presented in random order save that each word or picture was used at least once. Participants were then given a second set of instructions for stage 2. They were told that the response to the pictures was the same (right button for pictures of men, left for pictures of women) but that the response to the words had changed and they should now press the left button for sex words and right button for not-sex words. Fifty-six trials were then presented. For each block, the first eight trials were regarded as practice trials and were not analyzed.

The male-neutral sex IAT and female-neutral sex IATs were as identical as possible to the male–female sex IAT. The major change was that one of the set of gender pictures was replaced by a set of neutral images chosen for their lack of any sexual connotation and low valence and low arousal ratings on the IAPs and included pictures of natural scenes and man-made objects (nos: 5220, 5260, 5300, 5390, 5660, 5875, 7000, 7020).

#### Priming Task

The equipment and stimuli (pictures and words) were the same as used for the IATs. Each trial consisted of a fixation cross (1000 ms), the priming image (200 ms) and then the target word which remained until the participant responded. The participants completed 8 practice trials (all using a neutral prime not used in the data collection phase) followed by 120 trials (40 male primes, 40 female primes, 40 neutral primes) with 20 trials of each prime being followed by a sex word and 20 of each by a not-sex word. Trials were presented in random order that used a different seed for each participant.

### Data Analysis

Data from any participants scoring greater than 30% errors were removed for that task. Data from each of the tasks were trimmed by removing RTs less than 300 ms and greater than 2000 ms. The mean RT for each of the conditions was calculated for each participant. The data from the means from all tasks tended to show a non-normal distribution typical of reaction time tasks. These data were transformed by a reciprocal transform. The transformed data showed no departure from a normal distribution (Kolmogorov–Smirnov) and were used for the statistical analyses. However, the raw data are used for the figures and tables for clarity of interpretation.

For each task (IAT and Priming), the overall effects were examined via the appropriate analysis of variance (ANOVA). Interaction effects were examined by calculating IAT and priming effects as the difference in speed of RTs between the conditions (e.g., RT_male-sex_ − RT_female-sex_) and then comparing the magnitude of these effects across the appropriate contrasts via* t*-tests.

## Results

### Feeling Thermometer

For the explicit ratings, the data were bimodal and hence nonparametric statistics (Wilcoxon signed rank tests) were used. For the Feeling Thermometer, androphilic women gave more highly favorable ratings to sex with men than sex with women (95.6 vs. 19.7; *Z* = 7.58, *p* < .001; *g* = 4.12, 95% CI [3.43, 4.89]) while gynephilic women showed the opposite bias (14.2 vs. 93.0; *Z* = 5.45, *p* < .001; *g* = 3.20, 95% CI [2.35, 4.14]). The ambiphilic women showed slightly higher ratings to sex with men (82.7 vs. 72.7; *Z* = 2.10, *p* = .04 (two-tailed); *g* = 0.41, 95% CI [0.01, 0.83].

### Implicit Association Test

#### Female vs. Male Sex Implicit Association Task

Data from one ambiphilic participant were corrupted and could not be used. Four androphilic and two gynephilic participants were removed due to excessive error rates (> 30%). To calculate the reliability of the measure, trials were split into two via an odd–even split and the IAT bias effect (see below) was calculated for each set. The IAT bias scores were correlated and then corrected for loss of trials due to splitting via the Spearman-Brown prophecy equation. Reliability of the measure was excellent (*r* = .85).

**Fig. 1:**
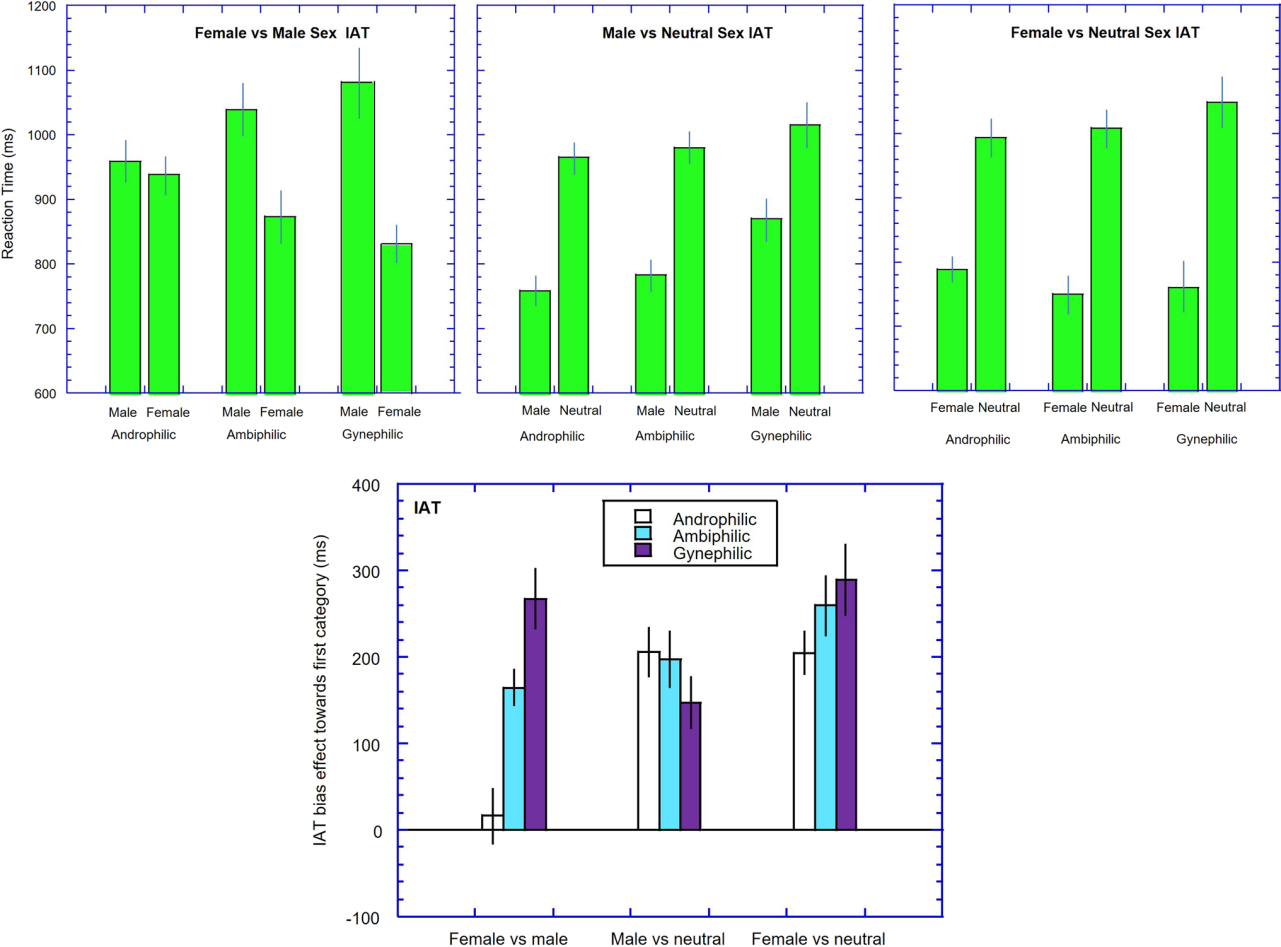
Upper panels. Reaction times (ms) in the implicit association tests (IATs) are plotted as a function of concept pairings for the three groups of women (androphilic, ambiphilic, and gynephilic) and the three version of the IAT (Left: female vs. male; Middle: male vs. neutral: Right: female vs. neutral). Error bars represent ± 1 standard error of the mean (SEM). Lower panel. The IAT effect (e.g., RT_men_sex_ – RT_women_sex_) is plotted for the three groups and for the three tasks. Error bars represent ± 1 SEM

The results are illustrated in Fig. 1 (top left panel). A 2 (target: male or female images paired with sex) by 3 (group: androphilic, ambiphilic, gynephilic) mixed ANOVA showed an effect of sexual target (*F*(1, 159) = 79.51, *p* < .001, *η*_p_^2^ = 0.33, 95% CI [0.22, 0.43]) but not of group (*F*(2, 159) = 0.09, *p* = .92). The interaction was significant (*F*(2, 159) = 22.35, *p* < .001, *η*_p_^2^ = 0.22, 95% CI [0.11, 0.32]).

To understand these effects, we calculated a IAT bias effect towards women (RT_male-sex_ − RT_female-sex_). Positive scores indicate greater attraction to women than men and are illustrated in Fig. 1 (bottom panel).

The bias effect for the androphilic women did not differ from zero (*d* = 0.08, *p* = .49), whereas the bias effect was large for ambiphilic women (*d* = 0.91, *p* < .001) and for gynephilic women (*d* = 1.12, *p* < .001). The bias effect in the androphilic group differed from both the ambiphilic women (*g* = 0.77, *p* < .001) and the gynephilic women (*g* = 1.17, *p* < .001). The bias effect was also larger for the gynephilic women than the ambiphilic women (*g* = 0.49, *p* = .02).

#### Male vs. Neutral Sex Implicit Association Task

Data from one androphilic participant were corrupted and could not be used. Four androphilic, one ambiphilic, and one gynephilic participants were removed due to excessive error rates (> 30%).

Reaction times are shown in Fig. 1 (top middle panel). A 2 by 3 mixed ANOVA showed a significant effect of target (*F*(1, 159) = 189.40, *p* < .001, *η*_p_^2^ = 0.54, 95% CI [0.44, 0.62]), no main effect of group (*F*(2, 159) = 2.63, *p* = .08, *η*_p_^2^ = 0.03), and a significant target-group interaction (*F*(2, 159) = 7.22, *p* = .03, *η*_p_^2^ = 0.08, 95% CI [0.01, 0.17]).

To understand these effects we calculated a difference score (RT_neutral-sex_ − RT_male-sex_)—hence, positive scores indicate greater attraction to male than neutral stimuli. These are shown in Fig. 1 (bottom panel). All three groups showed IAT effects such that they were faster in the male-sex condition than the neutral-sex condition (androphilic: *d* = 1.27; ambiphilic: *d* = 1.17; gynephilic: *d* = 0.76: all *p*s < .001). The bias score for the androphilic group was larger than for the gynephilic group (*g* = 0.34, *p* = .04 (one-tailed)) but no other group comparisons were significant.

#### Female vs. Neutral Sex Implicit Association Task

Data from two gynephilic participants were corrupted and could not be used. Four androphilic and one gynephilic participants were removed due to excessive error rates (> 30%).

Reaction times are shown in Fig. 1 (right panel). A 2 by 3 mixed ANOVA showed a significant effect of target (*F*(1, 158) = 285.4, *p* < .001, *η*_p_^2^ = 0.64, 95% CI [0.56, 0.71]), no main effect of group (*F*(2, 158) = 0.08, *p* = .92, *η*_p_^2^ = 0.001), and a significant target-group interaction (*F*(2, 158) = 4.18, *p* = .02, *η*_p_^2^ = 0.05, 95% CI [0.001, 0.12]).

To understand these effects we calculated a difference score (RT_neutral-sex_ − RT_female-sex_)—hence, positive scores indicate greater attraction to female than neutral images. These are shown in Fig. 1 (bottom panel). All three groups showed IAT effects such that they were faster in the female-sex condition than the neutral-sex condition (androphilic: *d* = 1.01; ambiphilic: *d* = 1.52; gynephilic: *d* = 1.45: all *p*s < .001). The bias score for the androphilic group was smaller than for the gynephilic group (*g* = 0.42, *p* = .04), but not the ambiphilic group (*g* = 0.28, *p* = .12). The ambiphilic and gynephilic groups did not differ (*g* = 0.16, *p* = .47).

### Priming Task

Data from three androphilic participants were removed, one due to missing data and two due to excessive error rate (> 30%). To calculate the reliability of the measure, trials were split into two via an odd–even split and the Prime bias effect (see below) was calculated for each set. The bias scores were correlated and then corrected for loss of trials due to splitting via the Spearman-Brown prophecy equation. Reliability of the measure was poor (*r* = .48).

**Fig. 2:**
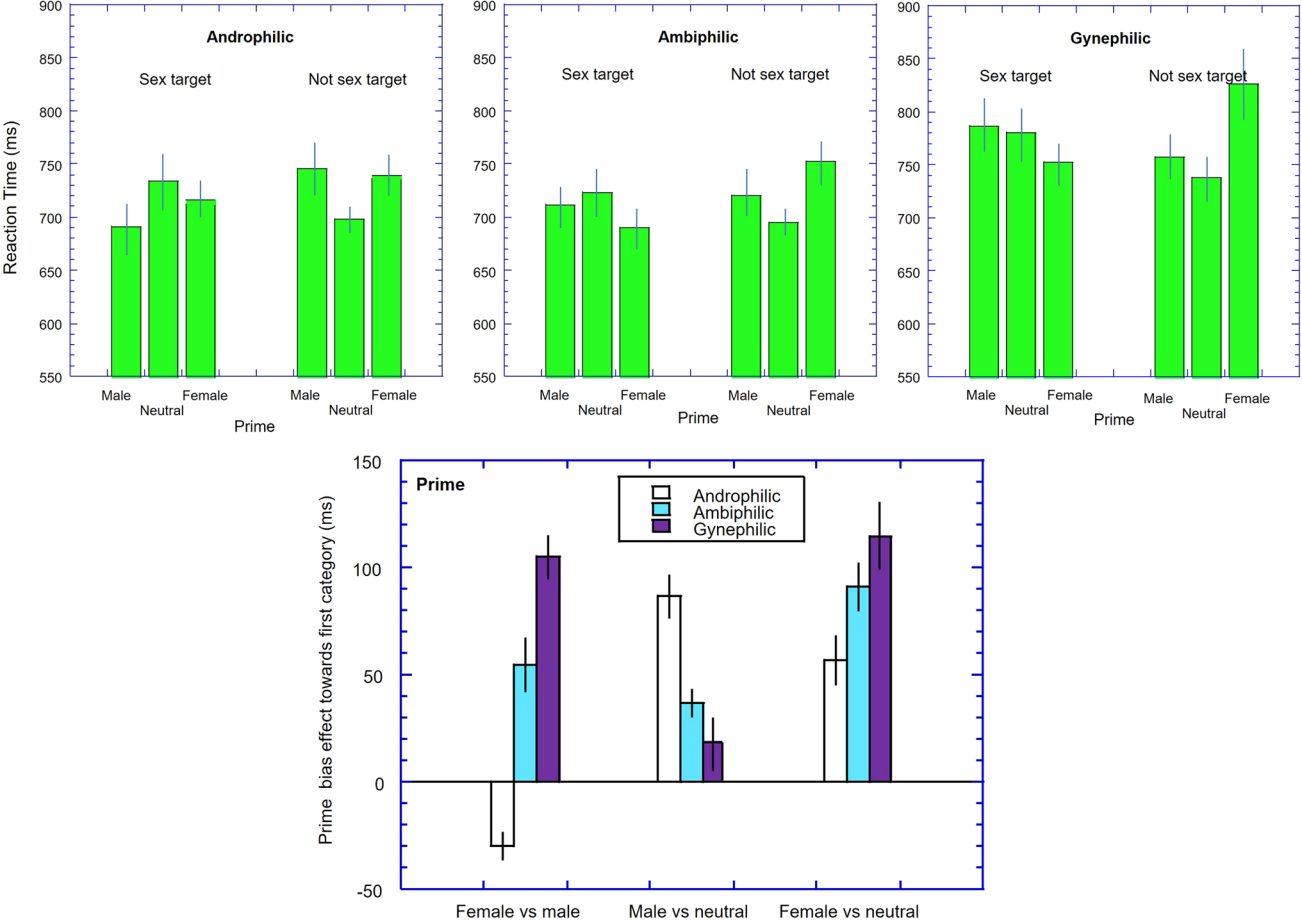
Upper panels. Reaction times (ms) in the prime task are plotted as a function of concept pairings for the three groups of women (Left: androphilic; Middle: ambiphilic; Right: gynephilic). Error bars represent ± 1 SEM. Lower panel. The prime effect (e.g. (RT_men/sex_ + RT_women/not sex_) − (RT_women/sex_ + RT_men/not sex_)) is plotted for the three comparisons and the three groups. Error bars represent ± 1 SEM

The data for the RTs are plotted in Fig. 2 (top panels). The data were subject to a 2 (target: sex, not-sex) by 3 (prime: male, neutral, female picture) by three (group: androphilic, ambiphilic, gynephilic) mixed ANOVA. This analysis revealed a main effect of prime *F*(2, 164) = 7.30, *p* < .001, *η*_p_^2^ = 0.04, but no main effect of target, *F*(1, 164) = 2.57, *p* = .11, *η*_p_^2^ = 0.02, or group, *F*(2, 164) = 2.7017.25, *p* = .07, *η*_p_^2^ = 0.03. There was no significant interaction between prime and group, or target and group (*F*s < 1) but there was a prime by target interaction, *F*(2, 328) = 39.90, *p* < .001, *η*_p_^2^ = 0.20. Crucially, the expected three-way interaction was significant (*F*(4, 328) = 9.09, *p* < .001, *η*_p_^2^ = 0.10). To understand this interaction, we first calculated prime bias effects. For instance, to compare priming by male and female images we pooled the trials indicative of sexual priming by male stimuli (male-sex trials with the female-not sex trails), and subtracted the trials indicative of sexual priming by female stimuli (male-not sex and female-sex trials)—bias index = ((RT_male_sex_ + RT_female_notsex_) − (RT_female_sex_ + RT_male_notsex_))—note higher scores indicate a greater priming between female and sex. Similar bias scores were also calculated for the male vs neutral primes, and for the female vs neutral primes. These indices are shown in Fig. 2 (bottom panel) and were analyzed separately.

#### Male vs. Female Comparison

A one-way between groups ANOVA showed a significant effect of group, *F*(2, 164) = 17.25, *p* < .001, *η*_p_^2^ = 0.17. For the androphilic group, the priming effect was consistent with a bias towards male and sex (*d* = − 0.25, *p* = .03). However, the ambiphilic group had stronger priming from the female primes than the male primes (*d* = 0.50, *p* = .001), as did the gynephilic women (*d* = 0.70, *p* < .001). The priming effect for the androphilic group differed from ambiphilic group (*g* = 0.72, *p* < .001) and the gynephilic group (*g* = 1.02, *p* < .001). The priming effects for the ambiphilic and gynephilic groups did not differ significantly (*g* = 0.39, *p* = .07).

#### Male vs. Neutral Comparison

A one-way between groups ANOVA showed a significant effect of group, *F*(2, 164) = 7.48, *p* < .001, *η*_p_^2^ = 0.08. The male primes (compared to neutral) produced a large effect size for the androphilic group (*d* = 0.79, *p* < .001), a small effect for the ambiphilic group (*d* = 0.33, *p* = .03), but no priming effect for the gynephilic group (*d* = 0.09, *p* = .56). The priming effect was significantly greater for the androphilic group than the ambiphilic group (*g* = 0.46, *p* = .01) and the gynephilic group (*g* = 0.70, *p* < .001). The priming effects for the ambiphilic and gynephilic groups did not differ significantly (*g* = 0.23, *p* = .25).

#### Female vs. Neutral Comparison

A one-way between groups ANOVA showed a marginally a significant effect of group, *F*(2, 164) = 2.83, *p* = .06. The female primes (compared to neutral) produced a moderate effect size for the androphilic group (*d* = 0.48, *p* < .001), a moderate effect for the ambiphilic group (*d* = 0.67, *p* < .001), and a large effect for the gynephilic group (*d* = 0.75, *p* < .001). The priming effect was greater for the gynephilic group than the androphilic group (*g* = 0.44, *p* = .02) and but not for the ambiphilic group (*g* = 0.27, *p* = .14). The priming effect for the ambiphilic and androphilic groups did not differ significantly (*g* = 0.17, *p* = .43).

## Discussion

The study was the first to examine implicit sexual attractions in ambiphilic women using the IAT and the priming task (though see Kirby et al. [2021] for measure of sexual identity using the IAT). As expected, women who classed themselves as ambiphilic gave explicit reports of approximately equal sexual attraction to men and women, though with a slight bias towards attraction to men. However, their implicit cognitions clearly illustrated a sexual attraction bias towards women for both implicit tasks. The main aim of the study was to examine if the explicit and implicit measures of sexual attraction would be in accord. This was not the case. The second aim was to examine if, instead, their implicit attractions lay between those of androphilic and gynephilic women. The overall pattern of results (see bottom panels of Figs. 1 and 2) support this notion of the response of ambiphilic women lying between those of androphilic and gynephilic women, though not all comparisons achieved statistical significance.

The present study was driven by the idea that the processing of sexual images proceeds via a dual-route (as has been suggested for many areas of psychology—Gawronski & Creighton, 2013) with an early automatic appraisal of the stimulus, and a later controlled process generating sexual responses (Janssen et al., 2000). Our tasks were designed to tap this early automatic appraisal stage via the automatic associations generated by the (brief) presentation of the images of men and women, and therefore suggests that for ambiphilic women these early appraisals are category specific with a bias towards images of females producing greater sexual attraction than images of males.

The response of ambiphilic women to male vs female images has been studied with other indirect tests. As discussed in the Introduction, measures using viewing time have generally found that viewing times to male and female models were approximately equal for ambiphilic women (Ebsworth & Lalumière, 2012; Lippa, 2013, 2017; Rullo et al., 2010). However, we argue that these viewing time measures are likely to be heavily dependent on the later “controlled” processes rather than the early automatic appraisal processes, and hence should be more in-line with peoples’ self-reported sexual attractions (including those of ambiphilic women). In order to assess these earlier components of visual attention, Dawson et al. (2017) presented two images (one of a man, one of a woman) and examined eye movements. They found that the first movement of the eyes was more likely to be towards the female image in ambiphilic women, whereas the total viewing time to each image was approximately equal to the two images. They too interpret their results in terms of the dual-process theory where the early automatic appraisal is biased to female stimuli, whereas the later controlled processes are not category specific in ambiphilic women. Further, Snowden et al. (2023) examined covert movements of attention (those without eye movements) using a dot-probe task where two images (one female, one male) are presented either side of a fixation mark and then replaced by a target at the location of one of the images. They found that targets appearing at the location of the female image were responded to more quickly in comparison to targets at the location of the male image in ambiphilic women (see Snowden et al., 2016 for results relating to androphilic women). Given the brief presentation (200 ms) of the images, the results were interpreted as tapping the early automatic component of attention (longer presentations were not used) which supports the results of Dawson et al. (2017) that their initial movement of attention is towards female, rather than male, images in ambiphilic women.

The results of the present study appear to support results from physiological recordings of genital response to images of males and females. Chivers et al. (2015) examined these responses due to the presentation of both visual film clips and auditory narratives. For exclusively androphilic women, responses were approximately equal to male stimuli and female stimuli. However, for women with any degree of gynephilia, including ambiphilic women, genital responses were greater to the female stimuli (see also Bouchard et al., 2015; Timmers et al., 2015). While such genital responses to stimuli lasting several seconds are likely to be influenced by both early automatic processes as well as later controlled processes, the present data show that even the early automatic associations/evaluations of simple visual images are indicative of a bias towards female stimuli in women reporting ambiphilic attractions.

The results of the present study, however, appear to differ from those of Kirby et al. (2021) who measured sexual identity using an IAT (see Introduction). Their results showed that ambiphilic women (and men) responses were indicative of a bisexual identity. The present results indicate greater implicit sexual attraction to females. It should be noted, however, that sexual orientation/identity and sexual attractions/associations are not synonymous (Sell, 1997) and there are clear examples, discussed in the Introduction (e.g., Chivers et al., 2004), where sexual identity and sexual arousal do not correspond. Hence, the present results of a greater implicit sexual attraction to females than males, and findings of greater sexual arousal to female images than male images (Bouchard et al., 2015; Chivers et al., 2015; Timmers et al., 2015) in ambiphilic women does not call into question that women who report a bisexual identity do indeed have a bisexual identity (Feinstein & Galupo, 2020). The present results, those from early movement of attention (Dawson et al., 2017; Snowden et al., 2023), and those from measures of genital responses, merely show that implicit sexual attraction, early attention allocation, and genital arousal does not correspond with self-reported sexual identity in ambiphilic/bisexual women (as well as the previous evidence showing a lack of correspondence in androphilic/heterosexual women). It would clearly be of interest to look at whether implicit measures of sexual identity, as measured by the technique of Kirby et al. (2021), show a greater correspondence with implicit sexual attractions as measured in the present studies.

On the whole, the results from the two measures (IAT and priming) gave a very similar pattern of results (see comparison of bottom panels of Figs. 1 and 2). So while the two tasks may tap different stages of associations, with the prime task assessing the associations to the actual exemplars and the IAT assessing associations to the concept categories (Olson & Fazio, 2003), it appears that these associations are highly similar for sexual associations in these samples. However, there may be one noteworthy exception. The two measures gave somewhat different results for the androphilic women, with the IAT showing no category-specific sexual attraction, but the priming task indicating some degree of category specificity towards the male stimuli. However, we note that this category specificity for the priming task was not apparent in an earlier study (Snowden & Gray, 2013). The reasons for this difference between the studies is not clear, but may relate to the specific exemplars used in the studies (as the priming task is thought to be sensitive to specific exemplars used (Fazio & Olson, 2003), or possible differences in the make-up of the samples. Further work will be needed to see if these differences are reliable.

The IAT has been criticized on several grounds (for a discussion, see Jost, 2019; Schimmack, 2021). First, is that, in its conventional form, it is a “relative” measure—in this case of sexual associations to male vs female images. So a bias towards say women could arise from a strong association between sex and women or from a weak association between sex and men. We attempted to address this issue by also constructing IATs that only had either male or female images and compared these to a “neutral” alternative. The results (see Fig. 1 bottom panel) showed that androphilic women had approximately equal sexual biases to men and to women, the ambiphilic women had a greater effects on the female-neutral IAT compared to the male-neutral IAT, with the gynephilic women showing the same, but more exaggerated, effect. Hence, the results from these two IATs parallel those from the female-male IAT and suggest that increasing levels of gynephilia produce an increasing implicit sexual attraction to women, and a reducing implicit sexual attraction to men. However, such a conclusion rests on there being equal sexual attraction (or lack thereof) for the neutral stimuli.

Second, the finding of associations between sexual words and female images is not necessarily the same as actual sexual attraction. One might associate sex with a particular item (for instance, “condoms”) without having any sexual attraction to the item. Hence, the interpretation of any individual result should be treated with caution. However, we believe that the “pattern” of results in this and other studies using the IAT technique appears more consistent with sexual attraction than some other interpretation. Further, the close correspondence between the results from the IAT technique (Snowden & Gray, 2013; Snowden et al., 2008, 2020) and direct measurement of genital arousal (Bouchard et al., 2015; Chivers et al., 2004, 2007, 2015; Peterson et al., 2010; Timmers et al., 2015) appears supportive of such an interpretation. Similar arguments apply to results using the priming technique.

It might also be argued that the results from the female-neutral IAT and male-neutral IATs suggest that all women show a degree of bisexuality as their sexual associations to both male and female images were greater than to neutral stimuli (with a similar pattern for these comparisons for the prime task). Such an interpretation is consistent with previous results examining both genital responses and pupil dilation responses (Rieger et al., 2016). Similar experiments to those of the present report (Snowden et al., 2020) also show that men (both gynephilic, ambiphilic, and androphilic) also show greater sexual associations to both male and female images than to the neutral images and so could be interpreted as suggesting that all men are bisexual to some degree.

### Limitations and Future Directions

Like most studies requiring volunteers to take part in studies of sexuality and be exposed to sexual stimuli, it seems likely that those volunteering may not be truly representative of the population with more liberal sexual attitudes and greater sexual experience and interest in sex (Wolchik et al., 1983), though it is unclear why this might affect the groups differentially. Further, the people tested in the present study tended to be young adults and from a Western culture. It would be of interest to extend such studies to other age groups and cultures with different rates of non-heterosexuality (though variations appear quite small—Rahman et al., 2020), in particular those with different sociopolitical views of non-heterosexual people (Flores, 2019). Such studies have already proved valuable in assessing theories and prevalence of non-heterosexuality in males (Colledani & Ciani, 2021) and the ease of use of such implicit cognitive measurements, in contrast to the use of measures of genital arousal, including on-line delivery, make these techniques useful for large-scale screening studies of populations.

### Conclusion

The present data show that ambiphilic women have greater implicit attraction to female stimuli than male stimuli, and therefore these implicit sexual attraction differ from their explicit judgements and their sexual identity. There results do not challenge the legitimacy of their sexual orientation in any way, but serve to show that early automatic evaluations of stimuli can be different from those arrived at by controlled and deliberate processes.

## Data Availability

The data are available at Snowden, Robert (2023), “Sex IAT and Prime Tasks_Women January 2023”, Mendeley Data, V1, https://doi.org/10.17632/fw654f83b4.1.

## Code Availability

The code for the studies are available from the lead author on request, save that the stimuli used are copyrighted (International Affective Picture System (Lang et al., 1997)).
